# Interfacial Properties of Bamboo Fiber-Reinforced High-Density Polyethylene Composites by Different Methods for Adding Nano Calcium Carbonate

**DOI:** 10.3390/polym9110587

**Published:** 2017-11-07

**Authors:** Cuicui Wang, Yu Xian, Lee M. Smith, Ge Wang, Haitao Cheng, Shuangbao Zhang

**Affiliations:** 1 International Centre for Bamboo and Rattan, Beijing 100102, China; cuicui124@163.com (C.W.); wangge@icbr.ac.cn (G.W.); 2 Beijing Key Laboratory of Wood Science and Engineering, Beijing Forestry University, Beijing 100083, China; 3 The College of Forestry of Shanxi Agricultural University, Jinzhong 030801, China; xianyu1919@126.com; 4 Department of Mechanical and Energy Engineering, University of North Texas, Denton, TX 76207-7102, USA; leemiller.smith27@gmail.com

**Keywords:** nano calcium carbonate (CaCO_3_), bamboo fiber (BF), impregnation modification (IM), blending modification (BM), high-density polyethylene (HDPE), interfacial properties

## Abstract

The focus of this study was to observe the effect of nano calcium carbonate (CaCO_3_) modification methods on bamboo fiber (BF) used in BF-reinforced high-density polyethylene (HDPE) composites manufactured by extrusion molding. Two methods were used to introduce the nano CaCO_3_ into the BF for modification; the first was blending modification (BM) and the second was impregnation modification (IM). In order to determine the effects of the modification methods, the water absorption, surface free energy and interfacial properties of the unmodified composites were compared to those of the composites made from the two modification methods. The results revealed that the percentage increase in the weight of the composite treated by nano CaCO_3_ decreased and that of the IMBF/HDPE composite was the lowest over the seven months of time. The results obtained by the acid-base model according to the Lewis and Owens-Wendt- Rabel-Kaelble (OWRK) equations indicated that the surface energy of the composites was between 40 and 50 mJ/m^2^. When compared to the control sample, the maximum storage modulus (*E′_max_*) of the BMBF/HDPE and IMBF/HDPE composites increased 1.43- and 1.53-fold, respectively. The values of the phase-to-phase interaction parameter *B* and the *k* value of the modified composites were higher than those of the unmodified composites, while the apparent activation energy *E_a_* and interface parameter *A* were lower in the modified composites. It can be concluded that nano CaCO_3_ had an effect on the interfacial properties of BF-reinforced HDPE composites, and the interface bonding between IMBF and HDPE was greatest among the composites.

## 1. Introduction

Bamboo fiber (BF) has become an ecologically viable substitute for wood fiber and presents great potential for various applications in the polymer composite industry. BF-reinforced polymer composites have been extensively explored because of the properties of BFs [1,2]. Similarly to other cellulosic fiber-reinforced polymer composites, the critical concerns regarding BF in reinforced composites is the poor interfacial adhesion between the BF and matrix, which results from the hydrophilic nature of BF that acts in opposition to the hydrophobic nature of the polymer matrix. The conflict between hydrophilic and hydrophobic components restricts the properties of the composites, leading to poor fiber partition and dispersion throughout the composites [3,4]. The interfacial properties are a key area of focus when studying plant fiber-reinforced polymer composites. At the interface, a high adhesive strength can transfer stress between the fiber and polymer, but a low adhesive strength will see the composite damaged (layered or broken) when it sustains an external load. Hence, it is essential to improve the matrix-fiber adhesion, which will ensure effective stress transfer in the composite, whereas the transmission efficiency depends mainly upon the interface bonding between the fiber and matrix.

There are two types of modification methods that can be used to improve interface bonding in fiber-reinforced polymer composites: the first uses a hydrophobic group to replace the hydroxy group on the fiber’s surface, thus making the BF more compatible with the hydrophobic polymer [5,6,7]. The second method, which is considered to be more effective, is to reduce the number of hydroxy group on the fiber surface via forming a chemical bond with the matrix [8,9,10]. Shi et al. [11] reported that inorganic nanoparticle impregnation is an effective way to improve the interface bonding between the natural fibers and polymer matrix. The inorganic nanoparticles deposited into the cell wall and on the surface, serving as nucleation sites, have a potential to improve the crystalline formation in the polymer matrix, which may provide strong static electric attractive forces to the non-polar polymer surface; hence the interface compatibility between the fibers and the matrix may be improved. Among the inorganic nanoparticle modification methods, nano calcium carbonate (CaCO_3_) modification has been developing considerable prospects because of the low cost, nano-scale, and ultra-fine solid structure of CaCO_3_, as well as the high economic value of the process. Thus, in previous research, BF-reinforced composites were treated with nano CaCO_3_ in situ modification, for which CaCO_3_ particles were successfully deposited into the BF [12,13]. The modification was studied using multiple analytical methods and parameters (e.g., environmental scanning electron microscopy (ESEM), atomic force microscopy (AFM), contact angle (CA) and tensile test), and it was observed that there was significant improvement in the interface compatibility, tensile strength and modulus of elasticity (MOE) of the composites. In a later study [14], impregnation modification (IM) was used to treat BF with nano CaCO_3_, and although this saw an increase in tensile properties of single BFs and composites, it was inferior to CaCO_3_ in situ modification. The optimal conditions for nano CaCO_3_ IM were obtained, and with this method, the technology used for fiber modification can be simplified.

Presently, the interfacial property of BF-reinforced polymer composites is studied on the basis of the interface bonding theory. The characterization methods for the interfacial property mainly include a fiber pullout test, a micro-bond test, a single-fiber fragmentation test, a micro-indentation test, AFM, static mechanics SEM, a CA test, dynamic mechanical analysis, and so forth [15]. In this study, one of the aims was to establish a connection between the macroscopic property and interface performance, and the surface free energy calculated by the CA and dynamic mechanical analysis was used. The surface free energy is a measurement of the energy on the material’s surface, which is also the macro embodiment of micro-mechanics at the atomic or molecular level of the material. The surface free energy not only evaluates the macroscopic property of the material, but it can also associate with the microscopic phenomena of the material [16]. Additionally, the structure of the composites, the compatibility of each component in the composites, and the relationships between mobility of the polymer chains and performance of the composites can be reflected by dynamic mechanical analysis [17]. Hong et al. [18] noted that dynamic mechanical flexural properties exhibit sensitivity to the interfacial adhesion between the lignocellulose and the polymer matrix. Ibarra and Panos [19] also reported that the interpretation of the dynamic properties and other parameters (e.g., glass transition temperature, apparent activation energy and interface performance parameter, etc.) could help to explain the existence of such interactions.

In this work, BF-reinforced high-density polyethylene (HDPE) composites were made from extrusion molding, and the BFs were treated by nano CaCO_3_ using blending modification (BM) and IM. The interfacial property was characterized by a CA test and dynamic mechanical analysis (DMA); moreover, the water absorption of the composites was also studied. The aim of this study was to build a relationship between the macro-performance and the micro-characteristics of the composites and to explore the interface enhancements of nano CaCO_3_, which may provide new theory and practice foundations for studying inorganic particle-reinforcing bamboo plastic composites for practical applications.

## 2. Materials and Methods

### 2.1. Materials

BF was obtained from Guizhou CHTH paper Co., Ltd., Guizhou, China. HDPE with a density of 0.945 g/cm^3^ (DGDK-3364, 0.075 g/min melt flow index at 190 °C and 2.16 kg, 22.1 MPa tensile strength) was purchased from Guangzhou Zhangmutou Plastic Co., Ltd., Guangzhou, China. The lubricant polyethylene wax (PE-wax; LPE-F4) was provided by Beijing Chemical University Keyi Fine Chemical Co., Ltd., Beijing, China. Maleic anhydride-grafted polyethylene (MAPE; 2909A^#^, 2.5–7 g/10 min melt-flow index at 190 °C and 2.16 kg) was obtained from Shenzhen Hualin Chemical Co., Ltd., Shenzhen, China. Nano CaCO_3_ with a grain diameter of 15–40 nm (NCCa40) was supplied by Beijing Boyu Gaoke New Material Technology Co., Ltd., Beijing, China. Ethylenediamine tetraacetic acid disodium salt (EDTA-2Na, analytically pure) serving as the dispersant was purchased from Beijing Huabo Stand Biological Analysis Technology Co., Ltd., Beijing, China.

### 2.2. Nano CaCO_3_ Modification Process

In this study, three types of BF/HDPE composites were produced. The first samples were made from unmodified BF, which served as a control, while the other two samples were made from BF treated with nano CaCO_3_ IM and BM.

IM: At 25 °C, 100 g of BF was dissolved into 2 L of deionized water for 0.5 h at 60 rpm; then 1.7 g of EDTA-2Na and 20 g of nano CaCO_3_ were introduced to the mixture, which was mixed for 25 min. The suspensions were washed on a 200-mesh nylon net with deionized water and were then air dried. Modified bamboo fibers (MBFs) were obtained and preserved at a constant temperature and humidity of 23 °C and 50% relative humidity (RH). BM: An equivalent content of nano CaCO_3_ was used to blend with BF in accordance with the loading of nano CaCO_3_ in the process of IM.

### 2.3. Composite Manufacture through Extrusion Molding Process

BF and IMBF were dried at 103 °C in an oven until the moisture content was reduced to less than 2 wt %.

**Figure 1:**
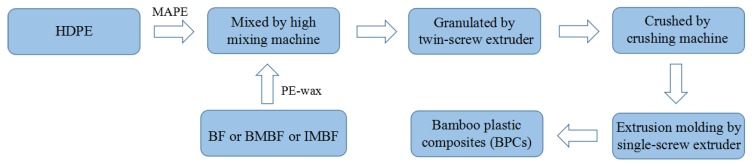
The process flowchart of extrusion molding.

BF, IMBF or BMBF with a mass fraction of 30 wt %, PE-wax with a mass fraction of 1 wt %, HDPE with a mass fraction of 65 wt % and MAPE with a mass fraction of 4 wt % were mixed together. The composites were obtained when the mixture was granulated by a twin-screw extruder (SD-30) (Shanghai Sunlight Plastics Machinery Manufacturing Co., Ltd., Shanghai, China) and crushed by a crushing machine (KCP150) (Beijing Kaichuang Tonghe Technology Development Co., Ltd., Beijing, China), obtaining granules. The granules were placed in a single-screw extruder (30 × 25) (Shanghai Sunlight Plastics Machinery Manufacturing Co., Ltd., Shanghai, China), where they were subjected to melt mixing and then water cooling to produce the final composites. BF/HDPE, BMBF/HDPE and IMBF/HDPE composites with a size of 15,000 mm × 30 mm × 4 mm (5000 mm × 30 mm × 4 mm each, length by width by thickness) were prepared by extrusion molding. In addition, the barrel temperatures used by the single-screw extruder were 160, 170, 180 and 175 °C, respectively. The process flow diagram of extrusion molding is shown in Figure 1.

### 2.4. Water Absorption Tests

Nine composites with dimensions of 76.2 mm × 25.4 mm × 4 mm prepared from the BF-, BMBF- and IMBF-reinforced composites (three for each) were used for the weight-gain rate measurements for water absorption testing. The water absorption test was carried out in accordance with the American Society for Testing and Materials (ASTM) D570-98 standard for the determination of the weight gain rate of the composites. The percentage increase in weight during immersion was calculated to the nearest 0.01% as follows:

(1) $$WA=\frac{W_{w}-W_{c}}{W_{c}}\times 100$$

where *WA* is the percentage increase in weight (%), *W_w_* is the wet weight (g), and *W_c_* is the conditioned weight (g).

### 2.5. Contact Angle Tests

The CAs of the composites were measured using an optical CA measurement instrument (Dataphsics OCA20). A charge-coupled device (CCD) with 7X objective and 25 fps was used to record the whole process of a liquid drop (deionized water, diiodomethane, formamide and glycerol) contacting with the sample. The CAs were calculated using the ellipse method and nine positions of each composite were tested in an ambient environment of 22 ± 1 °C and 50 ± 10% RH.

### 2.6. The Calculation of Surface Free Energy

There are many theoretical methods to calculate the solid surface energy, such as the Zisman method, the Fowkes method, the Owens-Wendt-Rabel-Kaelble (OWRK) method, the extended Fowkes method, acid-base theory formula, the theory of equilibrium state, the Schultz method, and so forth. These theoretical methods were obtained on the basis of Young’s equation. The polarity effect between the measured solid and liquid is not considered by the Fowkes method when calculating the surface energy [16]. Thus, the OWRK method and acid-base theory formula were suitable for this study because of the hydroxy group of BFs; the surface free energy of the composites was obtained through the OWRK method and acid-base theory formula according to the measured CA values. Table 1 shows the surface free energy parameters of the control liquid.

The OWRK method [20,21] divides the interfacial tension of each phase into two parts, that is, the dispersion component *γ^d^* and the polar component *γ^p^* (Equation (2)). Provided that the interaction between the polarity and dispersion force exists in the two phases of liquid and solid, the solid-liquid surface free energy *γ_SL_* can be represented as the geometric average function of *γ^d^* and *γ^p^* [22] (Equation (3)). Furthermore, the relationship between the CA *θ* formed on the solid surface and the solid surface free energy can be expressed by Young’s equation [23] (Equation (4)):

(2) $$\gamma =\gamma^{d}+\gamma^{P}$$

(3) $$\gamma_{SL}=\gamma_{L}+\gamma_{S}-2\sqrt{\gamma_{L}^{d}\gamma_{S}^{d}}-2\sqrt{\gamma_{L}^{P}\gamma_{S}^{P}}$$

(4) $$\gamma_{S}=\gamma_{SL}+\gamma_{L}\cos\theta$$

where *γ_L_* is the surface tension of the liquid; *γ_L_^d^* and *γ_L_^p^* are the dispersion component and polar component of the liquid surface tension, respectively; *γ_S_* is the solid surface free energy; and *γ_S_^d^* and *γ_S_^p^* are the dispersion component and polar component of the solid surface free energy, respectively.

By combining Equations (3) and (4), Equation (5) is obtained:

(5) $$\frac{\gamma_{L}(1+\cos\theta )}{2\sqrt{\gamma_{L}^{d}}}=\sqrt{\frac{\gamma_{L}^{P}}{\gamma_{L}^{d}}}\times \sqrt{\gamma_{S}^{P}}+\sqrt{\gamma_{S}^{d}}$$

Therefore, if the CA *θ*, *γ_L_*, *γ_L_^d^* and *γ_L_^p^* of at least two liquids are known, according to Equation (5) and the graphing method, $\sqrt{\frac{\gamma_{L}^{P}}{\gamma_{L}^{d}}}$ and $\frac{\gamma_{L}(1+\cos\theta )}{2\sqrt{\gamma_{L}^{d}}}$ serve as the *x* axis and *y* axis, respectively, allowing the slope and intercept of the line to be used to calculate *γ_S_^p^* and *γ_S_^d^*. Thus, the solid surface free energy *γ_S_* can be obtained by Equation (2).

Acid-base theory [24,25,26,27] converts *γ^d^* to the Lifshitz-van der Waals component *γ^LW^* and divides *γ^p^* into the Lewis acid component *γ*^+^ and Lewis alkali component *γ*^−^. Then Equation (3) can be converted to Equation (6):

(6) $$\gamma_{SL}=\gamma_{S}+\gamma_{L}-2\sqrt{\gamma_{S}^{LW}\gamma_{L}^{LW}}-2\sqrt{\gamma_{S}^{+}\gamma_{L}^{-}}-2\sqrt{\gamma_{S}^{-}\gamma_{L}^{+}}$$

By uniting Equations (4) and (6), Equation (7) is then derived:

(7) $$\gamma_{L}(1+cos\theta )=2\sqrt{\gamma_{S}^{LW}\gamma_{L}^{LW}}+2\sqrt{\gamma_{S}^{+}\gamma_{L}^{-}}+2\sqrt{\gamma_{S}^{-}\gamma_{L}^{+}}$$

According to Equation (7), if the CA *θ*, *γ_L_*, *γ_L_^LW^*, *γ_L_*^+^ and *γ_L_*^−^ of at least three liquids are known, *γ_S_^LW^*, *γ_S_*^+^ and *γ_S_*^−^ can be calculated; thus it was possible for *γ* to be obtained.

### 2.7. Dynamic Mechanical Property Tests

The dynamic mechanical properties of the composites were measured in the dual-cantilever mode (Q800 DMA, Thermal Analysis Instruments, New Castle, DE, USA) at frequencies of 1, 2, 5, 10 and 20 Hz. The dimensions of the sample were 60 mm × 14 mm with a thickness of 4 mm. Tests were conducted in the range of −20 to 120 °C at a heating rate of 2 °C/min with a strain amplitude of 25 μm (ASTM standard D7028-07^ε1^).

## 3. Results and Discussion

### 3.1. Water Resistance

**Figure 2:**
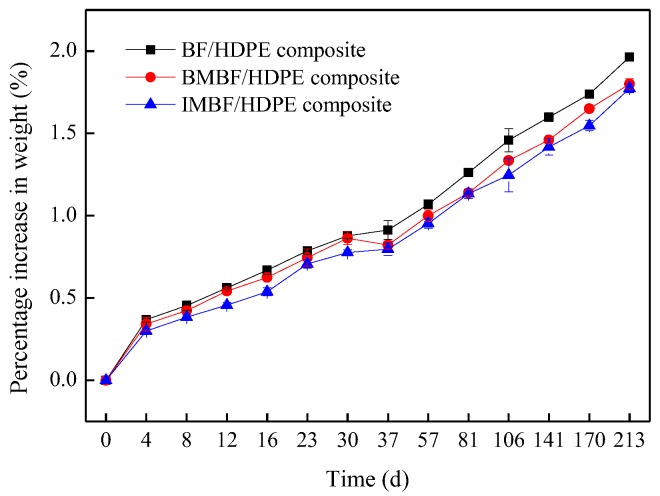
The percentage increase in weight of composites.

The results of the water resistance properties showed that nano CaCO_3_ treatment reduced the percentage increase in the weight of composites (Figure 2). This is because nano CaCO_3_ successfully adhered onto the BF surfaces using either nano CaCO_3_ modification method and also reduced the contact between the distilled water and BF. Nano CaCO_3_ also decreased the number of hydroxy group [28], thus improving the water resistance. Furthermore, the CA of CaCO_3_ was 12.1° (Table 2), which indicated that CaCO_3_ was hydrophilic. As the precipitate, CaCO_3_ absorbed little water, and no swelling phenomenon was observed from submersion in distilled water. Therefore, adding nano CaCO_3_ can increase the water resistance of the composites. It was observed that nano CaCO_3_ IM had a greater effect on the water resistance than BM, which was due to agglomerations of nano CaCO_3_ forming on the surface of the BF during the BM.

### 3.2. Surface Free Energy of Composites

Table 2 presents the results of static CAs, including the nano CaCO_3_, BF/HDPE, BMBF/HDPE and IMBF/HDPE composites. There were differences among deionized water, formamide, diiodomethane and glycerol for CAs of the BF/HDPE, BMBF/HDPE or IMBF/HDPE composites due to the different chemical constitutions of the various liquids.

According to the data listed in Table 2, the surface energy of the composites was calculated by the acid-base model according to both the Lewis and OWRK equations. The surface energy results of the BF/HDPE, BMBF/HDPE and IMBF/HDPE composites are summarized for comparison in Table 3 and Table 4. Whichever calculation method was used, the surface energy of composites was between 40 to 50 mJ/m^2^, which indicated that the composites belonged to the hydrophilic materials. The surface energy of the IMBF/HDPE composite was the greatest of all the composites, followed by the BMBF/HDPE composite. Clearly, nano CaCO_3_ increased the surface free energy of the composites, as the surface free energy of CaCO_3_ was greater when CaCO_3_ was adhered to the surface of the BFs, increasing their surface free energy. In addition, when the particle size of CaCO_3_ was small enough, the dispersity and CA both increased, resulting in an improvement of the adhesion strength.

### 3.3. The Dynamic Thermo-Mechanical Properties (Single-Frequency)

**Figure 3:**
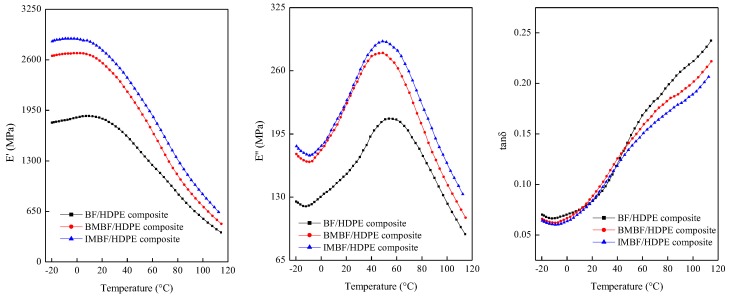
Single-frequency dynamic thermo-mechanical properties of composites (1 Hz).

Figure 3 illustrates the single-frequency dynamical thermo-mechanical properties of the composites with a temperature range from −20 to 120 °C. The storage modulus (*E*′) of the composites decreased as the temperature increased, indicating that the kinetic energy was low and the rigidity of the composites was high at low temperatures, while the HDPE matrix softening caused relaxation phenomena in the composites at high temperatures [29]. The *E*′ values of the composites treated with nano CaCO_3_ were higher than those of the BF/HDPE composite, which can be attributed to two reasons: the first is that the interface bonding could perform as a physical connection function; when the composites were under the cycle capacity, the BF and CaCO_3_ particles would bear the stress that was transferred from the matrix HDPE, which would restrict the movement of the molecular chain. The second reason is that the nano CaCO_3_ treatment could have decreased any voids caused by the BF, which in turn reduced any defects in the composite structure, causing the rigidity of the HDPE matrix and storage modulus of the composites to increase. Moreover, the storage modulus of the IMBF/HDPE composite was higher than that of the BMBF/HDPE composite; this was as a result of better interfacial compatibility caused by stronger connections in the molecular chain, leading to less slipping of the polymer matrix and hence a higher storage modulus [30], which was similar to the findings of our previous research on flexural properties [31].

It can be noted that the loss modulus (*E*′′) of the BF/HDPE, BMBF/HDPE and IMBF/HDPE composites increased in the plastic region and then decreased with increasing temperature in the rubbery region. The loss modulus was highest in the IMBF/HDPE and lowest in the BF/HDPE composite, which indicated that the mutual movement between the molecular chain of IMBF caused the greatest friction, and the elastic energy of the molecular motion was converted to more thermal energy. This led to the loss modulus of the IMBF/HDPE composite being highest at a macro level. The loss factor (tanδ) can measure the damping properties of the material and indicate the molecular motion in the material, which contributes at the interface to affect damping or energy dissipation [32,33]. As shown by the loss factor-temperature curve, the loss factor was different among the BF/HDPE, BMBF/HDPE and IMBF/HDPE composites, which indicates that nano CaCO_3_ had an effect on the loss factor of the composites. Moreover, the movement of the polymer segments was subjected to the CaCO_3_ and the heterogeneity in the crosslinking structure, affecting the damping characteristics of the composites and leading to improved elastic stiffness in the composites treated by nano CaCO_3_.

The phase-to-phase interaction parameter *B* was used to describe the effect on the dynamic mechanical properties of the composites by different methods of nano CaCO_3_ modification. The *B* value was calculated using Equation (8) [34,35]:

(8) $$\frac{E_{c}^{\prime }}{E_{m}^{\prime }}=\frac{1+1.5V_{f}B}{1-V_{f}B}$$

where *E_c_*′ and *E_m_*′ are the storage modulus of the composite and the HDPE matrix, respectively (MPa); *V_f_* is the volume fraction of the fiber (%); and *B* is the phase-to-phase interaction parameter.

Table 5 shows the glass transition temperature *T_g_*, loss factor tanδ, and *B* of the composites. When compared to the BF/HDPE composite, the *B* value of the composites treated by nano CaCO_3_ was increased, which showed that nano CaCO_3_ modification enhanced the phase-to-phase interaction of the composite. The greater the *B* value was, the greater the interface interaction was. This was because nano CaCO_3_ adhering to the surface of the BFs made the interface region form chemical bonds and changed the interfacial structure. Additionally, the *B* value of the IMBF/HDPE composite was higher than that of the BMBF/HDPE composite, indicating that the reinforcing effect of IM was superior to that of the BM.

### 3.4. The Dynamic Thermo-Mechanical Properties (Multiple-Frequency)

**Figure 4:**
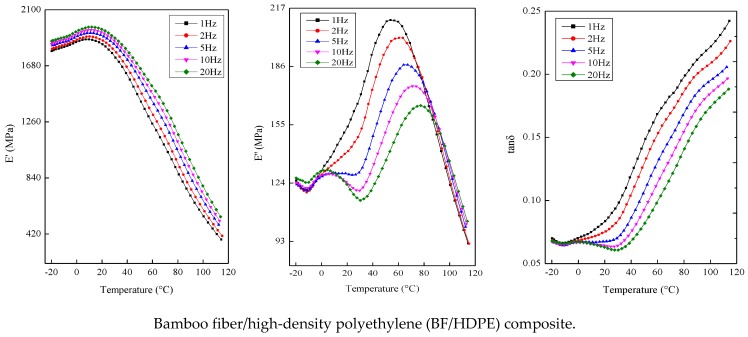

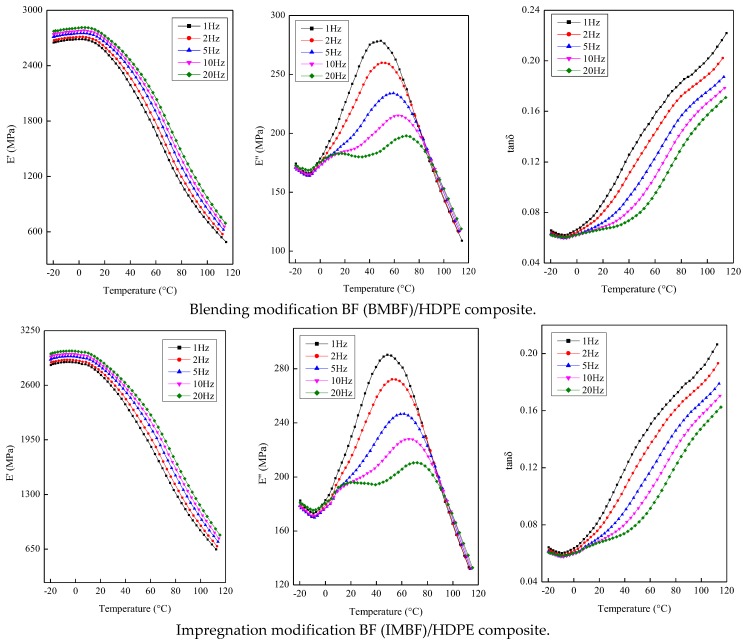
Multiple-frequency dynamic thermo-mechanic properties of composites.

Figure 4 shows the variations of the storage modulus (*E*′), loss modulus (*E*′′) and loss factor (tanδ) of the BF/HDPE, BMBF/HDPE and IMBF/HDPE composites, which were tested against frequency as a function of temperature. The storage modulus *E*′ of the composites was greater at high-frequency conditions than at low-frequency conditions. The plots of the storage modulus, the glass transition temperature (temperature at peak values of loss modulus *E*′′) and loss factor all moved to the high temperature zone as the test frequency increased. This can be attributed to two reasons: the first is that the inhibition of micro-Brownian motion of the HDPE molecular chain structure unit was enhanced by nano CaCO_3_. The second reason was due to the thermal hysteresis of the composites on the basis of time-temperature superposition; the increase in the frequency was equal to the increase in the heating rate. This meant that the faster the heating rate, the more serious the thermal hysteresis of the composites [30].

#### 3.4.1. The Effect on Storage Modulus *E*′ of Composite by the Test Frequency *f*

The correlation between the logarithmic frequency dependence of the storage modulus is described using Equation (9) [36] (Lagakos et al., 1986):

(9) $$E^{\prime }=k\mathrm{lg}f+b$$

where *E*′ is the storage modulus of composites; *k* is a value about the property of the composite, reflecting the frequency dependence of *E*′; *f* is the test frequency of the composite; and *b* is the piecewise function of the filling system.

By choosing temperatures (e.g., −20, 45, and 110 °C) from the plastic and rubbery regions, the *k* value was calculated according to Equation (9) by linear fitting. As can be seen from Table 6, in both the plastic region and rubbery region, the *k* values of the composites treated by nano CaCO_3_ were greater than those of the control sample. This trend confirmed that the nano CaCO_3_ modification influenced the frequency dependence of *E*′; in particular, the effect of IM was quite remarkable.

#### 3.4.2. The Apparent Activation Energy

The relation between *T_g_* and the frequency *f* can be described by the apparent activation energy *E_a_* and is calculated using Equation (10) according to the Arrhenius equation:

(10) $$f=A_{0}e^{-(E_{a}/RT)}$$

where *f* is test frequency (Hz); *A*_0_ is the pre-exponential factor (min^−1^); *E_a_* is the apparent activation energy (kJ/mol); *R* is the Planck gas constant, 8.314 × 10^−3^ kJ/(K·mol); and *T* is the test temperature (K).

On account of Equation (10), the computational formula of *E_a_* under the multiple frequency condition can be expressed by Equation (11):

(11) f1f2=e−(Ea/RTg1)e−(Ea/RTg2)

where *f*_1_/*f*_2_ is the level of the mobile factor, and *Tg*_1_ and *Tg*_2_ re the glass-transition temperatures corresponding to the frequencies *f*_1_ and *f*_2_, respectively.

On the basis of Equation (12), a linear regression was performed on the slope, and *E_a_* was calculated by plotting the natural log scale of the frequency *f* (Hz) against the inverse of the glass transition temperature *T_g_* (°C):

(12) $$E_{a}=-R\times \frac{d(\ln f)}{d(1/T_{g})}$$

**Figure 5:**
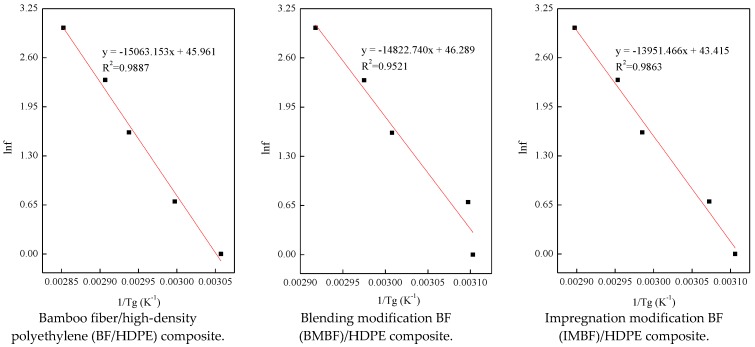
*E_a_* values of composites.

The resulting activation energies were 125.24, 123.24 and 115.99 kJ/mol for the BF/HDPE, BMBF/HDPE and IMBF/HDPE composites, respectively (Figure 5). Therefore it can be observed that both IM and BM had an effect on the *E_a_* value of the composites; this was due to the presence of CaCO_3_, which diminished the internal defects in the composites and improved the interfacial compatibility between BF and HDPE. Moreover, a lower energy barrier occurred during the relaxation of the IMBF/HDPE composites, which was in agreement with the better interfacial compatibility results of the IMBF/HDPE composite. Lv, Zhang, and Yu [37] reported that the smaller *E_a_* was, the less energy that was required for the molecular chain segments of the matrix during structural transformation. By the easy transformation of the composites, it can be proved that the interfacial properties of the composites were much improved.

### 3.5. The Interfacial Properties of Composites

The dynamic mechanics performance of a material can reflect its interfacial properties [17,29] and provides a convenient visual analysis method for characterizing interfacial properties, which also can be used for reference. The interface between the fibers and matrix can be quantified using a hybrid model of the loss factor according to previous research [33,38]. In order to determine the parameter of the interface performance *A*, Equation (13) was used:

(13) $$\tan\delta_{c}=V_{f}\tan\delta_{f}+V_{m}\tan\delta_{m}+V_{i}\tan\delta_{i}$$

where $\tan\delta_{c}$ is the loss factor of the composites; $V_{f}$ is the volume fraction of the fiber; $\tan\delta_{f}$ is the loss factor of the fiber; $V_{m}$ is the volume fraction of HDPE; $\tan\delta_{m}$ is the loss factor of HDPE; $V_{i}$ was the volume fraction of the interface; and $\tan\delta_{i}$ is the loss factor of the interface.

In order to compare the effect of different nano CaCO_3_ treatments on the state of adhesion between the phases, it was assumed that $\tan\delta_{f}$ ≈ 0; thus, the volume fraction of the interface was rather small, and Equation (13) was converted into Equations (14) and (15):

(14) $$\frac{\tan\delta_{c}}{\tan\delta_{m}}=(1-V_{f})(1+A)$$

(15) $$A=\frac{V_{i}}{1-V_{f}}\times \frac{\tan\delta_{i}}{\tan\delta_{m}}$$

Equation (14) can also be expressed as Equation (16), which was proposed by Ibarra and Panos (1998).

(16) $$A=\frac{\tan\delta_{c}}{\tan\delta_{m}}\times \frac{1}{1-V_{f}}-1$$

**Figure 6:**
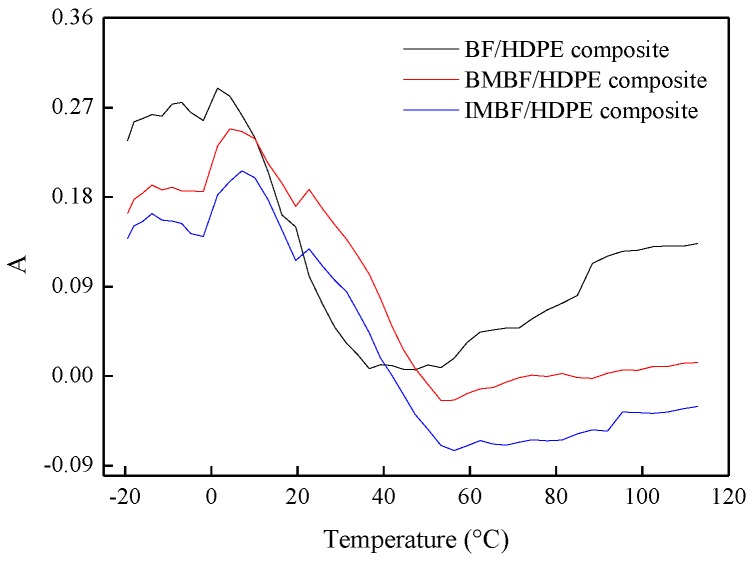
*A* values of composites.

Figure 6 showed how the *A* factor varied with the temperature for the BF/HDPE, BMBF/HDPE and IMBF/HDPE composites. The *A* value of the composites declined before the *T_g_* value. At the temperature exceeding *T_g_*, the *A* value of the composites treated by nano CaCO_3_ remained almost constant; this was because the interfacial force was saturated and the interfacial adhesion between the reinforcement and the matrix was rather good over this temperature interval. However, the *A* value of the BF/HDPE composite increased and remained quite different from that of the BMBF/HDPE and IMBF/HDPE composites, which was caused by the thermal contraction of the HDPE around the BF being released. It can be seen that the *A* value for the IMBF/HDPE composite was the lowest and the BF/HDPE composite was the highest within a range from *T_g_* to 120 °C. This indicated that the interactions between the IMBF and HDPE at the interface were strong, which means that the macro-molecular mobility around the surface of the IMBF was reduced in contrast with that of the matrix HDPE; thus $\tan\delta_{i}$ and *A* were reduced. A low value of the *A* factor was indicative of a high degree of fiber-matrix interaction or adhesion between the phases [38].

## 4. Conclusions

In this study, nano CaCO_3_ was introduced by IM and BM, and the BF/HDPE, BMBF/HDPE and IMBF/HDPE composites were prepared successfully by an extrusion molding process (the mass fraction of HDPE was 65%). Nano CaCO_3_ treatment reduced the percentage increase in the weight of the composites, and the reduction by IM was higher than that by BM. The acid-base model according to both the Lewis and OWRK equations was suitable for calculating the surface energy of the composites. Meanwhile, the change trend of the surface energy in the two methods was consistent, and the surface energy of the composites was between 40 and 50 mJ/m^2^. As deduced from the dynamic mechanical analysis (DMA) testing, the structural stability of the IMBF/HDPE composite was higher than that of the BMBF/HDPE and BF/HDPE composites under the conditions of a single frequency, while the *E′_max_* value of the BMBF/HDPE and IMBF/HDPE composites increased 1.43- and 1.53-fold, respectively, when compared to the control sample. The phase-to-phase interaction parameter *B* and *k* values of the composites treated by nano CaCO_3_ were higher than those of the control sample; in particular, the effect of IM was remarkable. When compared to the control sample, the *E_a_* value of the BMBF/HDPE and IMBF/HDPE composites decreased by 1.60% and 7.39%, respectively, which indicated that the interactions between the IMBF and HDPE at the interface were strong. It was observed that nano CaCO_3_ decreased the *A* value of composites in the temperature range of −20 to 120 °C, which confirmed this finding. Thus, the CaCO_3_ produced by IM and BM has an effect on the interfacial properties of BF/HDPE composites manufactured by the extrusion molding process, and the effect of IM was more apparent than that of BM.

## Figures and Tables

**Table 1:** Surface free energy parameters of control liquid (mJ/m^2^).

Types of liquid	$\gamma_{L}$	$\gamma_{L}^{d}$	$\gamma_{L}^{P}$	$\gamma_{L}^{\mathrm{LW}}$	$\gamma_{L}^{\mathrm{AB}}$	$\gamma_{L}^{+}$	$\gamma_{L}^{-}$
Deionized water	72.80	21.80	51.00	21.80	51.00	25.50	25.50
Diiodomethane	50.80	50.80	0.00	50.80	0.00	0.00	0.00
Formamide	58.00	39.00	19.00	39.00	19.00	2.28	39.60
Glycerol	64.00	34.00	30.00	34.00	30.00	3.90	57.40

**Table 2:** Static contact angles (°).

Sample	Deionized Water	Diiodomethane	Formamide	Glycerol
Nano CaCO_3_	12.1 (1.70)	24.1 (0.26)	12.6 (1.86)	—
BF/HDPE composite	81.53 (1.34)	39.53 (0.55)	67.60 (0.52)	80.23 (2.84)
BMBF/HDPE composite	80.06 (2.15)	39.50 (3.58)	68.85 (3.89)	83.60 (1.42)
IMBF/HDPE composite	81.03 (0.95)	37.85 (3.75)	70.30 (2.84)	59.95 (2.59)

**Table 3:** Surface energy calculated by acid-base model according to Lewis equation.

Sample	$\gamma_{S}$ (mJ/m^2^)	$\gamma_{S}^{\mathrm{LW}}$	$\gamma_{S}^{\mathrm{AB}}$	$\gamma_{S}^{+}$	$\gamma_{S}^{-}$
BF/HDPE composite	43.75	39.84	3.91	0.41	9.43
BMBF/HDPE composite	45.48	39.86	5.62	0.67	11.79
IMBF/HDPE composite	47.45	40.67	6.77	0.98	11.72

**Table 4:** Surface energy calculated by Owens-Wendt-Rabel-Kaelble (OWRK) equation.

Sample	$\gamma_{S}$ (mJ/m^2^)	$\gamma_{S}^{d}$	$\gamma_{S}^{P}$
BF/HDPE composite	42.80	39.84	2.96
BMBF/HDPE composites	43.28	39.86	3.42
IMBF/HDPE composites	43.63	40.67	2.96

**Table 5:** The dynamic mechanical data and *B* value of composites.

Sample	*Tg* (°C)	tanδ	*B*
BF/HDPE composite	53.93	0.1553	0.2138
BMBF/HDPE composite	49.11	0.1410	0.9348
IMBF/HDPE composite	48.78	0.1343	1.1020

**Table 6:** The *k* value of composites.

Sample	*k*		
	−20 °C	45 °C	110 °C
BF/HDPE composite	57.658	173.348	149.704
BMBF/HDPE composite	94.708	231.033	160.773
IMBF/HDPE composite	102.404	243.562	189.129
